# Differing Clinical Courses and Prognoses in Patients With Gastric Neuroendocrine Tumors Based on the 2010-WHO Classification Scheme

**DOI:** 10.1097/MD.0000000000001748

**Published:** 2015-11-06

**Authors:** Beom Su Kim, Young Soo Park, Jeong Hwan Yook, Sung Tae Oh, Byung-Sik Kim

**Affiliations:** From the Department of Surgery (BSK, JHY, STO, BSK); and Department of Pathology, Asan Medical Center, University of Ulsan College of Medicine, Seoul, Korea (YSP).

## Abstract

The aim of this study is to test the prognostic accuracy of the 2010-WHO classification for postsurgery survival in nonmetastatic gastric neuroendocrine tumor (NET) cases. Whether the 2010-WHO classification of NETs can predict relapse after surgical resection has not yet been established.

We selected 175 nonmetastatic gastric NET patients at Asan Medical Center, Seoul, Korea between 1996 and 2013. All tumors were classified using the WHO-2010 scheme.

Among 175 patients with gastric NETs, we diagnosed 39 cases as WHO grade 1, 13 cases as grade 2, 66 cases as grade 3 (neuroendocrine carcinomas; NECs), and 57 cases as mixed with adenocarcinoma. Patients with grade 3 had a lower relapse-free survival (RFS) and overall survival (OS) than those with WHO grade 1/2 and had a lower OS than patients with mixed type tumors. Patients with grade 1/2 had a better OS than patients with mixed type. There was no significant difference in RFS and OS between small and large cell type lesions. Among WHO grade 1/2 patients with ≤1 cm sized lesions, none exhibited lympho-vascular, perineural, mucosal, or submucosal invasion, and we detected no lymph node metastases or recurrences.

Our findings strongly suggest that WHO grade 3 behaves more aggressively than adenocarcinoma. Additionally, the survival of cases with large and small cell NEC was similar. Among WHO grade 1/2 patients who had ≤1 cm lesions, none exhibited lympho-vascular, perineural, mucosal, or submucosal invasion and all could be treated by endoscopic resection or minimally invasive surgery without node dissection.

## INTRODUCTION

The incidence of gastric neuroendocrine tumors (NETs) have increased over the past few decades^1,2^ although the incidence is lower than that of other gastrointestinal organs.^1,3^ Several guidelines of diagnosis and treatment of gastric NETs have been published due to the heterogeneity in biology and in clinical behavior of the tumor.^4,5^ Rindi et al^6^ classified gastric NETs into 3 subtypes of carcinoids; type 1 associated with chronic atrophic gastritis, which shows good prognosis;^7,8^ and type 2 associated with multiple endocrine neoplasia type 1 and Zollinger–Ellison syndrome, which usually shows good prognosis but with a few exceptions showing aggressive behavior.^9,10^ Type 3 refers to sporadic cases associated with the greatest malignancy potential, presenting the poorest prognosis among the 3 types.^7,11^ By contrast, Kim et al^12^ reported that regardless of the type, carcinoids that are not yet advanced can be effectively treated with minimal endoscopic or laparoscopic surgery.

In 2000, the World Health Organization (WHO)^13^ proposed a classification scheme for gastroenteric NETs (WHO-2000), and this was updated in 2010 (WHO-2010).^14^ In WHO-2000, pure NETs were classified into 1 of 3 tumor categories: well-differentiated endocrine tumors (WDETs) that exhibited benign behavior; well-differentiated endocrine carcinomas (WDECs) that showed low-grade malignant behavior; and poorly differentiated endocrine carcinomas (PDECs) that displayed high-grade malignant behavior.

In 2006, the European Neuroendocrine Tumor Society (ENETS) proposed a new grading system for NETs^5,15,16^ that was based on the Ki-67 index (grade 1, ≤2%; grade 2, 3%–20%; and grade 3, >20%) and the WHO-2010 adopted the Ki-67 labeling index and/or mitotic index for NETs. Grade 3 was classified into 2 types of high grade neuroendocrine carcinomas (NECs): large cell (LC) NECs and small cell (SC) NECs. Additionally, WHO-2010 defined mixed adenoneuroendocrine carcinomas (MANECs) that contained neuroendocrine cells (exceeding at least 30% of all tumor cells) mixed with nonendocrine components (usually adenocarcinoma structures).

Unfortunately, the 2010-WHO classification scheme has not yet been validated for its ability to predict relapse after surgical resection. Reports of the prognosis of gastric NETs based on the 2010-WHO classification are extremely rare.^17,18^ Therefore, we evaluated the prognostic accuracy of this classification scheme for survival after surgery for nonmetastatic gastric NET cases that were treated at a single institution.

## METHODS

Between 1996 and 2014, 175 patients were diagnosed with gastric NET at the Asan Medical Center in Seoul, Korea. We selected patients who did not have distant metastasis at the time of diagnosis and who underwent endoscopic resection or surgical resection of NETs (R0 resection). All tissues were reviewed by a pathologist and classified according to the WHO-2010 classification. Mixed type was defined as NETs mixed with adenocarcinoma. We evaluated risk factors for lymph node metastasis and prognostic factors. We evaluated the basic clinical features and survival data between WHO grades and AJCC stages. Additionally, clinical outcomes between grade 3 and mixed type, and between SC and LC type were evaluated. Relapse-free survival (RFS) was defined as the time from tumor resection to the earliest among the following outcomes: disease recurrence (local or metastatic), last follow-up without evidence of disease, or death without evidence of disease.

Numerical data were expressed as means with standard deviation using Student's *t*-tests. Risk factors were analyzed using the Chi-square test (univariate analysis) or a logistic regression model (multivariate analysis). Survival data were analyzed using the Kaplan–Meier method with the log-rank test (univariate analysis) or Cox proportional hazards regression (multivariate analysis). All statistical data were analyzed using SPSS 21.0 (SPSS Inc., Chicago, IL) software. *P-*values < 0.05 were considered to indicate statistically significant differences.

This study received approval from Asan Medical Center's Institutional Review Board (IRB).

## RESULTS

### Basic Clinicopathological Characteristics of Patients

The average follow-up period was 52.9 ± 41.5 months and the male-to-female ratio was 2.4:1. Table 1 shows the clinicopathological characteristics. Among the 175 patients with gastric NETs, we diagnosed 39 as WHO grade 1, 13 as type 2, 66 as type 3, and 57 as mixed type. Tumors were more commonly located in the lower to mid portion of the stomach. A total of 76.6% of patients underwent gastrectomy. Among 49 patients with tumor recurrences, 10 had recurrence in the remnant stomach.

**TABLE 1:**
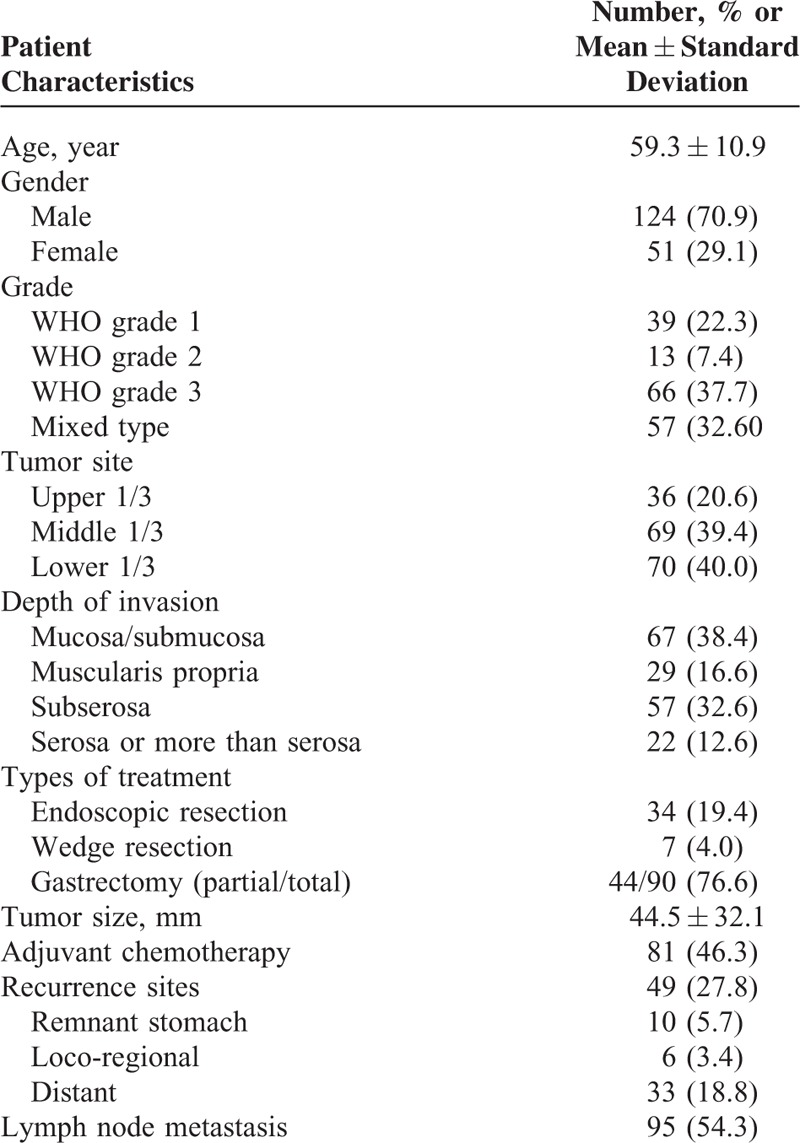
Clinicopathologic Characteristics of the Study Patients

### WHO Subgroup Analysis: Grades 1, 2, and 3

**FIGURE 1:**
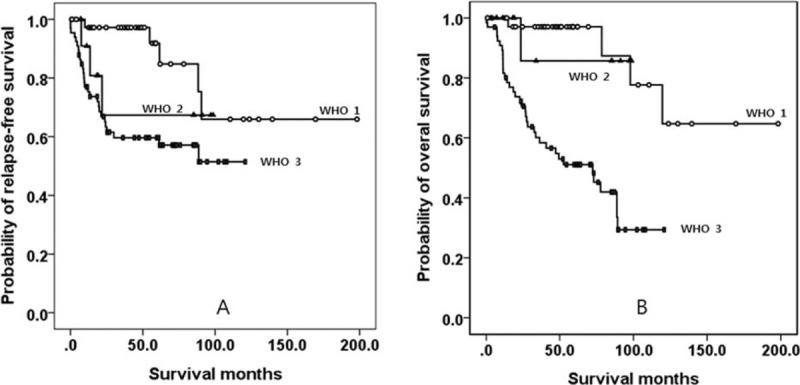
Kaplan–Meier survival curves of RFS (A) and OS (B) among patients WHO grade 1, 2, and 3 tumors. Patients with grade 3 lesions had a lower RFS and OS than those patients with grades 1 and 2. However, the RFS and OS for grades 1 and 2 were both high and not significantly different. OS = overall survival, RFS = relapse-free survival, WHO = World Health Organization.

There were distinct characteristic and clinicopathological differences among WHO grades 1, 2, and 3 (Table 2). Patients with grade 3 were older than patients with grade 1 and the tumor location of grade 3 was more predominantly in the lower portion of the stomach (*P* < 0.05). Notably, patients with grade 3 required more aggressive treatment and experienced both more lymph node metastases and tumor recurrence than those with grade 1 lesions (*P* < 0.05). However, there was no significant difference between grades 1 and 2 in age, gender, or lymph node metastasis (*P* > 0.05). In grade 1, 6 cases experienced tumor recurrence and all of these cases had recurrences at the remnant stomach. Additionally, 2 of 3 cases with grade 2 who experienced tumor recurrences had recurrences at the remnant stomach. By contrast, only 2 of 27 grade 3 cases with recurrences experienced recurrences at the remnant stomach, and 20 of 27 cases experienced distant recurrences. Figure 1 shows a Kaplan–Meier survival curve of RFS and overall survival (OS). Patients with grade 3 had a lower RFS and OS than the other 2 grades (*P* < 0.05). However, the RFS or OS of grades 1 and 2 were high and similar (*P* > 0.05).

**TABLE 2:**
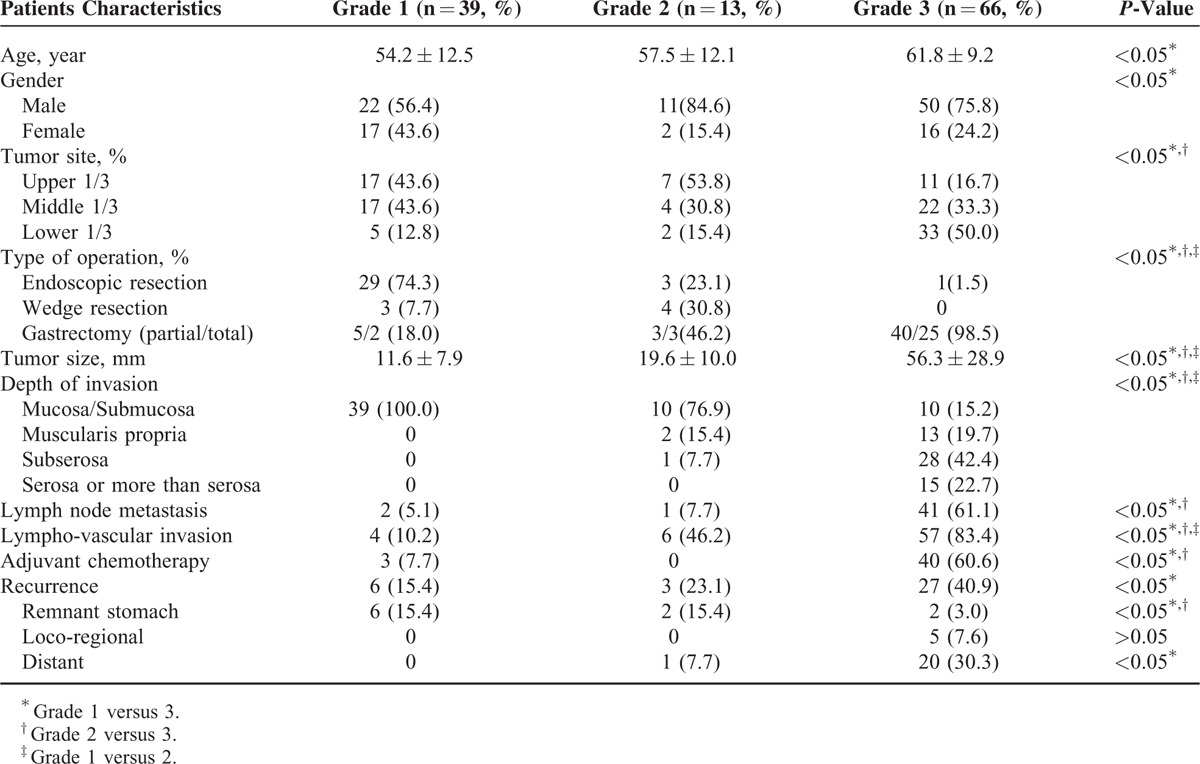
Matched-Pair Analysis of the Clinicopathological Properties of the Study Patients Based on the WHO Grading System

### Matched-Pair Analysis: Grade 1/2, Grade 3, and Mixed Type

**FIGURE 2:**
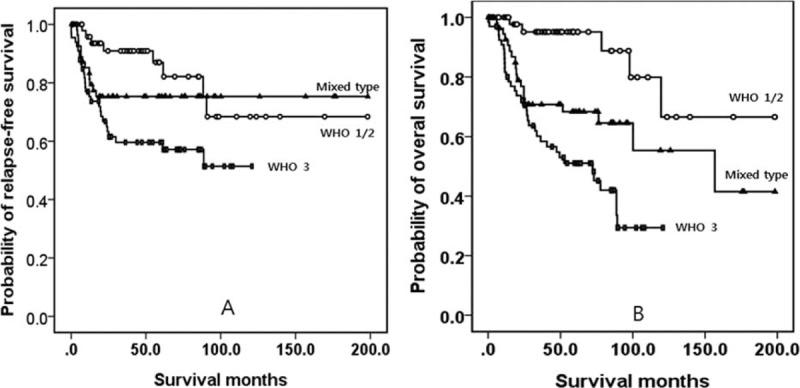
Kaplan–Meier survival curves for RFS (A) and OS (B) among WHO grade 1/2, 3, and mixed-type tumors. Patients with grade 3 tumors had a lower OS than those with mixed-type tumors. Patients with grade 1/2 tumors had a better RFS and OS than patients with grade 3, and also had a poorer OS than patients with mixed-type lesions. OS = overall survival, RFS = relapse-free survival, WHO = World Health Organization.

The results of the statistical analyses are summarized in Table 3. Although patients with grade 3 required more aggressive treatment, they experienced more tumor recurrence than those with mixed type lesions (*P* < 0.05). Although grade 1/2 cases mostly experienced tumor recurrence at the remnant stomach, grade 3 or mixed type cases mostly experienced tumor recurrences at a distant site (*P* < 0.05). Patients with grade 3 had a lower OS than those with a mixed type (Figure 2). However, patients with grade 1/2 were younger and had tumor locations that were more predominantly in the upper portion of the stomach than those of patients with grade 3 or mixed type lesions (*P* < 0.05). Additionally, patients with grade 1/2 had better overall clinical data (except for gender) than patients with grade 3 or mixed type lesions (*P* < 0.05). Finally, patients with grade 1/2 had a better RFS and OS than patients with grade 3 (Figure 2), and they also had a better OS than patients with mixed type lesions.

**TABLE 3:**
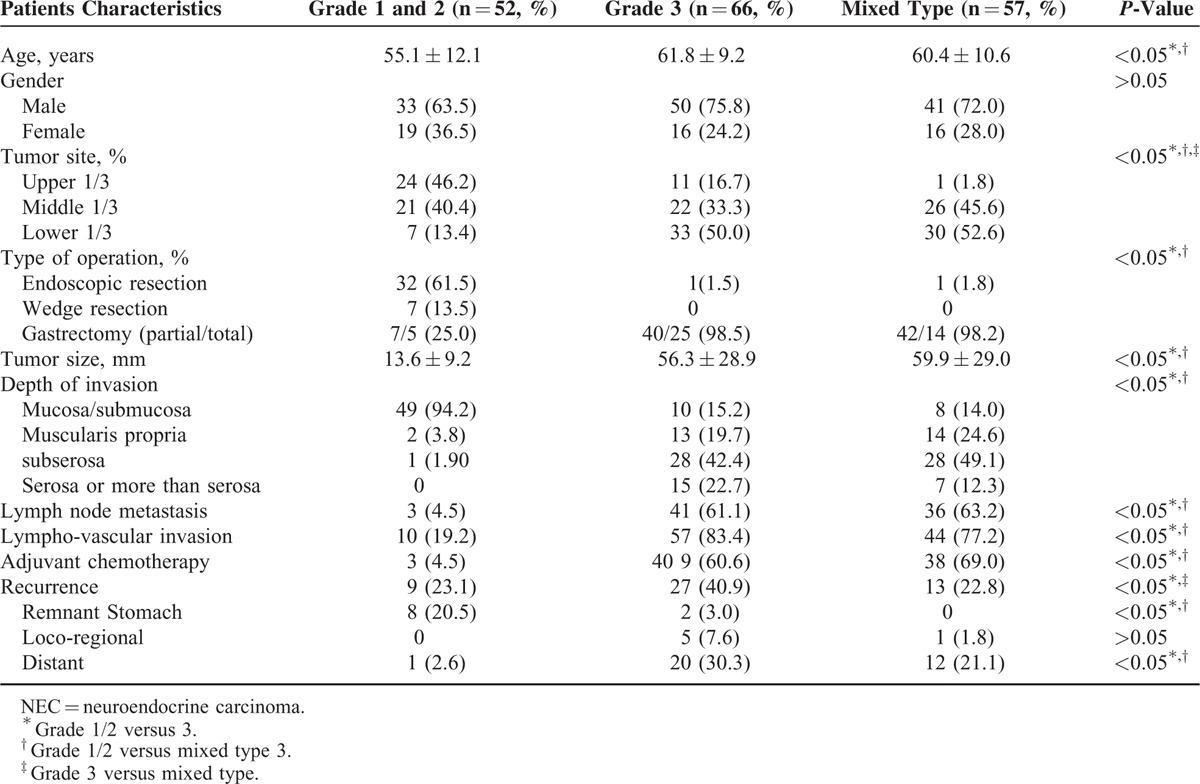
Matched-Pair Analysis of the Clinicopathological Properties of Grade 1/2, Grade 3 NEC, and Mixed Type Cases in the Study Series

### Matched-Pair Analysis of Small and Large Cell Type NEC

**FIGURE 3:**
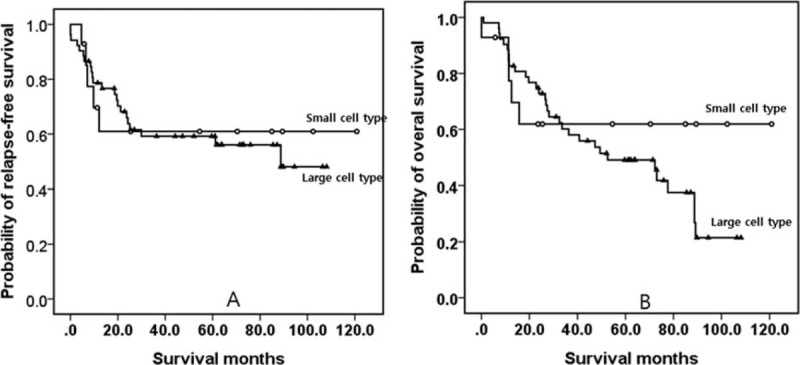
Kaplan–Meier survival curves of RFS (A) and OS (B) between small and large cell type tumors. There was no significant difference in the RFS or OS between the two. OS = overall survival, RFS = relapse-free survival.

There were no statistically significant differences among all categories, except for lympho-vascular invasion (Table 4). Figure 3 shows Kaplan–Meier survival curve analyses. There were no significant differences in RFS or OS between both groups (*P* > 0.05).

**TABLE 4:**
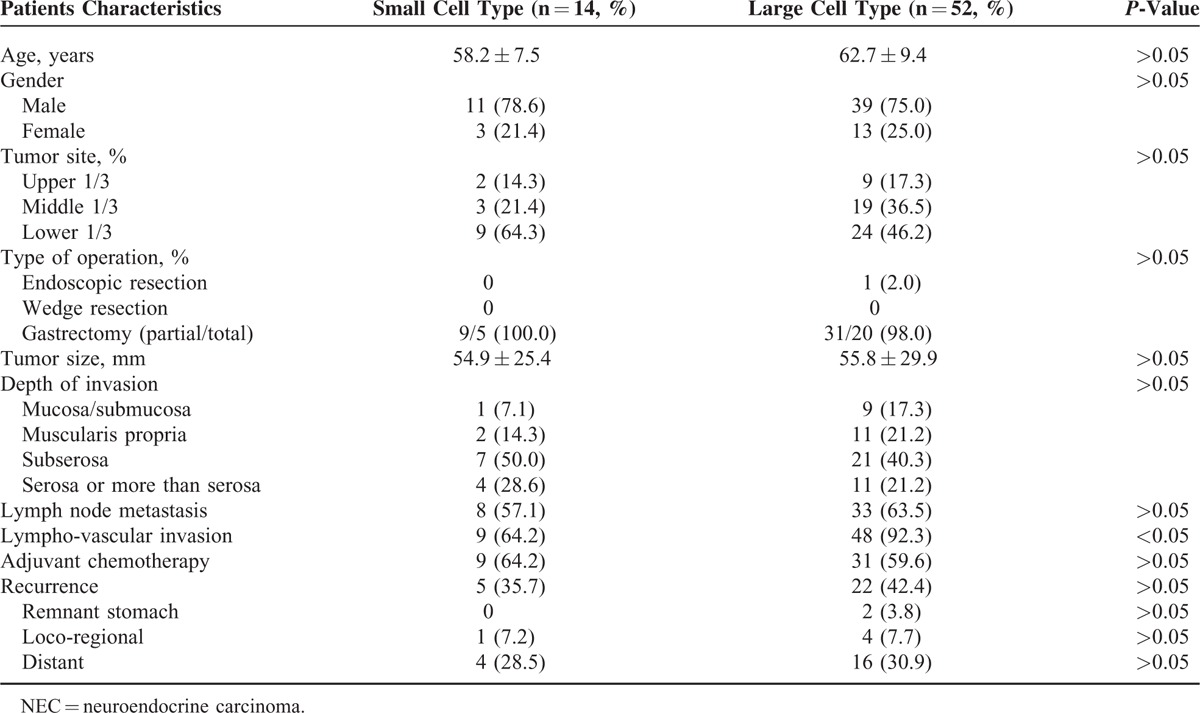
Matched-Pair Analysis of the Clinicopathological Properties of Small and Large Cell Type NEC in the Study Series

### Factors Influencing Patient Survival and Prognosis

Gender, WHO grade, tumor size, depth of invasion, lympho-vascular invasion, perineural invasion, and lymph node metastasis were found to influence prognostic factors based on univariate analysis using the Kaplan–Meier method with the log-rank test. The results of Cox proportional hazards analysis (Table 5) showed that WHO grade 3, lymph node metastasis, and deep depth of invasion were independent prognostic factors (*P* < 0.05). We evaluated Cox proportional hazards analysis as 2 groups: low grade (WHO grade 1/2) and high grade (WHO grade 3/mixed type). In the low-grade group, there was no multivariate prognostic risk factor. However, these results were similar to those shown in Table 5 for the high-grade groups (Table 6).

**TABLE 5:**
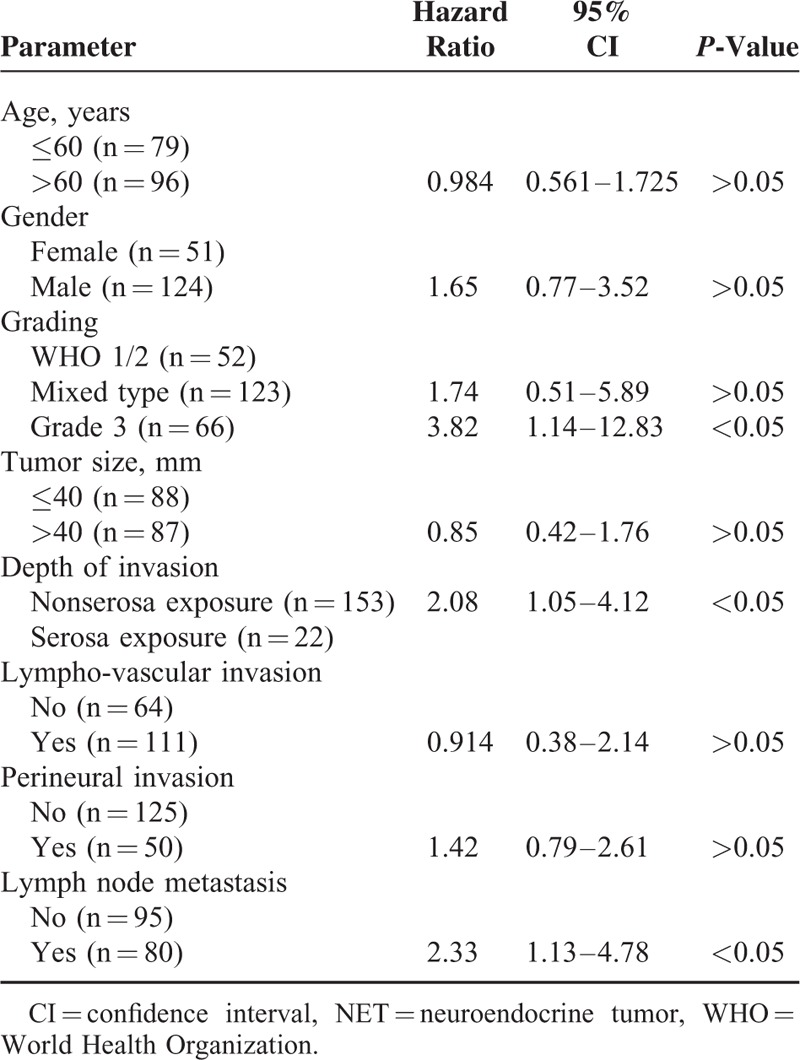
Multivariate Analysis of Prognostic Factors of all NETs

**TABLE 6:**
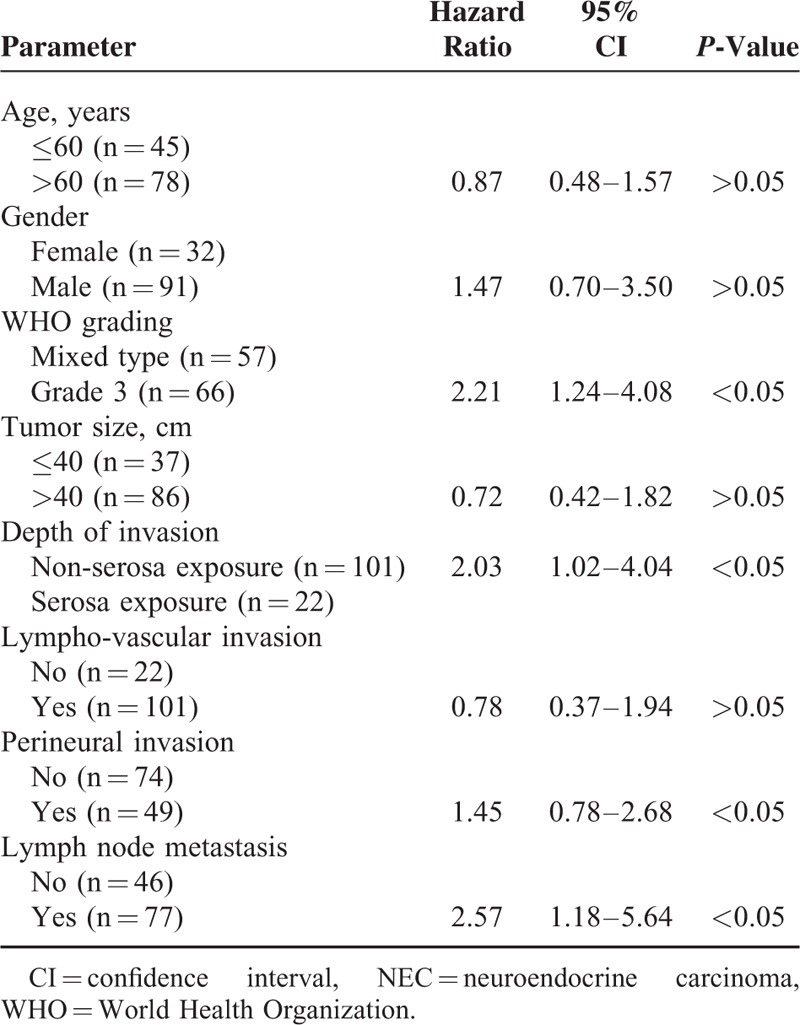
Multivariate Analysis of the Prognostic Factors for Grade 3 NEC/Mixed Type Cases

### Factors Influencing Lymph Node Metastasis

Lymph node metastasis is the most important risk factor after minimally invasive surgery without lymph node dissection. Lympho-vascular invasion and perineural invasion are independent risk factors that can influence lymph node metastasis in high grade NETs. Lymph node metastasis according to size distribution is summarized in Table 7. Grade 1 or 2 patients with ≤1 cm sized lesions had no evidence of lympho-vascular, perineural, no lymph node metastasis, or tumor recurrence.

**TABLE 7:**
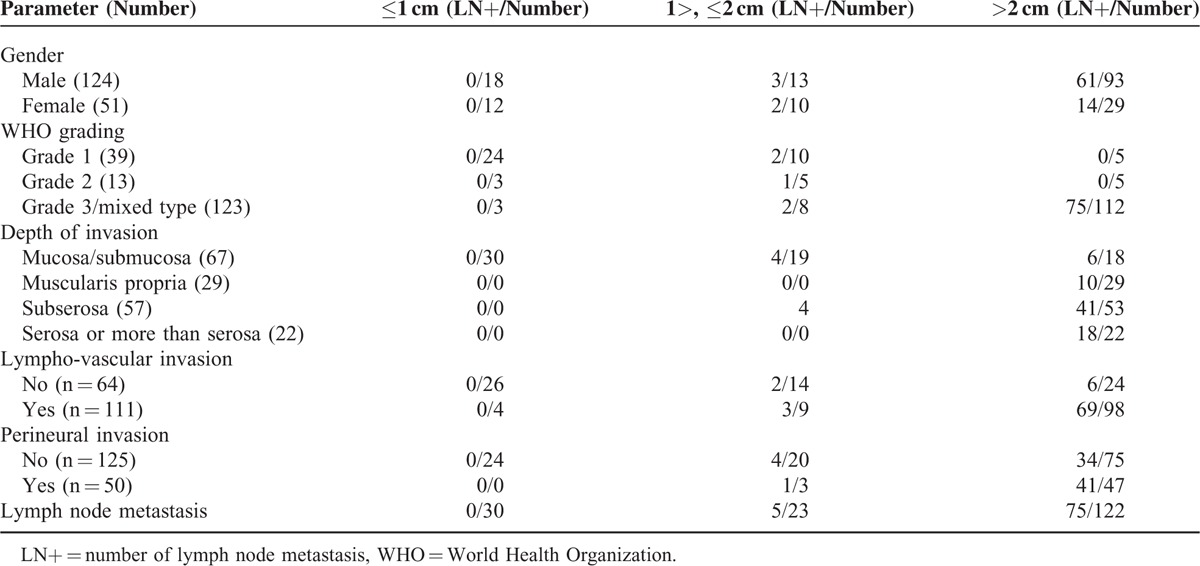
Lymph Node Metastasis According to Size Distribution

## DISCUSSION

The lack of a uniform staging system for gastric NETs has substantially disabled clinicians to predict the risk of recurrence and prognosis of patients suffering from this tumor. Previous clinical or pathologic classifications were not utilized worldwide because of their complexity and limitation in usefulness. Nevertheless, the AJCC, ENETS, and WHO staging classifications are interchangeably used, since they allow a little better stratification and risk assessment of gastric NETs. However, their ability to predict recurrence-free survival for gastric NETs has not yet been tested. In this present study, we analyzed the RFS outcomes of 157 patients with gastric NETs. This study validates the usefulness and limitation of the WHO-2010 scheme of gastric NETs to predict RFS after resection.

The use of the Ki-67 index and/or mitosis in the WHO and ENETS grading systems was validated for foregut and pancreatic NENs (PanNENs) by several studies and their biological relevance and power to discriminate among prognostic groups has mostly been confirmed.^19–23^ However, to the best of our knowledge, no previous report of the prognostic validation of gastric NETs according to both grading systems has been published. In our present study, we found that pure poorly differentiated NECs had worse outcomes than NETs mixed with adenocarcinoma. Additionally, LC NECs had outcomes that were similar to SC NECs. Therefore, NECs would be expected to behave more aggressively than adenocarcinomas. To date, similar results have not been described by other studies.

In the past few decades, many attempts have been made to uniformly treat gut endocrine tumors. Unfortunately, because of their rarity, no structured therapeutic approach has been developed, despite increased knowledge and awareness of this condition. In 2004 and 2012, ENETS reported guidelines for the treatment of gastrointestinal NETs according to 3 subtypes of classification.^5,6,24^ They recommended the adoption of the following treatment guidelines for small sized benign type 1 and 2 tumors that are within the submucosal layer can be observed or endoscopic resection can be performed. Benign type 1 and 2 tumors which extend to the muscularis or which have recurred need surgical resection. Malignant type 1 and 2 or recurrence after local resection needs radical gastrectomy. Type 3 and poorly differentiated tumors need radical gastrectomy. By contrast, Kim et al^12^ reported that irrespective of tumor type, typical carcinoids that are not yet advanced, could effectively be treated with minimal endoscopic or laparoscopic surgery, whereas NECs should be treated with radical gastrectomy, similar to carcinomas. The North American Neuroendocrine Tumor Society published guidelines in 2010^25,26^ and the National Comprehensive Cancer Network did so in 2011.^27^ These guidelines were both based on Rindi type and tumor size; however, they were not based on the WHO-2010 classification. Additionally, there are no guidelines for treatment according to the WHO classification. In this present study, we found that among grade 1 or 2 patients with ≤1 cm sized lesions, there were no cases of lympho-vascular, perineural, mucosal, or submucosal invasion, or any cases of lymph node metastasis. Therefore, these patients could be treated with endoscopic resection or minimally invasive surgery without node dissection.

We found distinctive differences in tumor recurrence in our current analyses. Patients with grade 1/2 tumors mostly experienced tumor recurrence at the remnant stomach, whereas patients with grade 3 or mixed type tumors mostly experienced tumor recurrence at a distant site. Therefore, endoscopy could be a more useful method to check for recurrence for WHO grade 1 or 2 lesions, while CT could be a more useful method to check for recurrence of grade 3 or mixed type lesions.

Our study had some limitations of note. This is a retrospective study. And, 2010-WHO classification is a histological classification on the basis of morphological criteria and the assessment of the proliferation fraction according to the ENETS scheme. This highlights that the histological classification alone is not sufficient to predict clinical evolution. Therefore, the accuracy of this paper still needs to be discussed. NETs of the stomach are very rare, so the statistical power of our analysis was limited by the relatively small number of patients. Recently, WHO-2010 defined mixed adeno-neuroendocrine carcinoma (MANEC) as a lesion that contains 30% of either component; however, we could not exactly determine the proportion of either component. Therefore, we defined NETs with any portion of adenocarcinoma as a mixed type. Finally, in our cohort, 61.5% of patients with grade 1 or 2 lesions received endoscopic resection and 98.4% of patients with grade 3 or mixed type lesions received gastrectomy. This difference between treatment methods could affect the sites of recurrence and lymph node metastasis.

In conclusion, we have found that cases of WHO grade 3 had poorer OS outcomes than NETs mixed with adenocarcinoma. Additionally, mixed type cases had a poorer OS than cases with WHO grade 1/2. These findings led us to speculate that NECs behave more aggressively than adenocarcinomas. We found that LC NECs had a similar RFS and OS as cases of SC NECs. Among WHO grade 1 or 2 patients with ≤1 cm sized lesions, no instances of lympho-vascular, perineural, mucosal, or submucosal invasion were noted, and no instances of lymph node metastases or recurrences were observed. Therefore, these patients could be treated with endoscopic resection or minimally invasive surgery without node dissection. We contend from this that endoscopy could be a more useful method for monitoring the recurrence of WHO grade 1 or 2 tumors, whereas CT could be a more useful method for monitoring the recurrence of grade 3 or mixed type lesions.
